# Surgery versus neoadjuvant chemoradiotherapy followed by surgery in locally advanced gastrointestinal tract cancers

**DOI:** 10.3389/fonc.2025.1631549

**Published:** 2025-09-10

**Authors:** Xuxing Ye, Zhangqiang Wu, Weijun Teng, Yili Zhang, Yanping Chen, Lin Sheng, Junmei Lin, Xiaobo Wang

**Affiliations:** ^1^ Department of Traditional Chinese Medicine, Jinhua Municipal Central Hospital, Jinhua, China; ^2^ Department of Surgical Oncology, Guang Fu Oncology Hospital, Jinhua, China; ^3^ Department of Gastroenterology, Jinhua Municipal Central Hospital, Jinhua, China; ^4^ Department of Health Management Center, Affiliated Jinhua Hospital, Jinhua Municipal Central Hospital, Jinhua, China; ^5^ Department of Pulmonary and Critical Care Medicine, Jinhua Municipal Central Hospital, Jinhua, China; ^6^ The Fourth School of Clinical Medicine, Zhejiang Chinese Medical University, Hangzhou, China

**Keywords:** neoadjuvant-applied chemoradiotherapy, postoperative complications, gastrointestinal tract cancer, GI cancer treatment, multimodal technique

## Abstract

**Background:**

Gastrointestinal tract cancer is still prevalent in the world. Localized GI cancer treatment has greatly relied on surgery, even for locally advanced diseases.

**Aim and objectives:**

The goal of this study was to assess the prognosis of direct surgery and neoadjuvant chemoradiotherapy and surgery in patients with locally advanced GI tract cancers.

**Materials and methods:**

A cross-sectional study was done on patients diagnosed with locally advanced GI cancers who were treated at Zhejiang Jinhua Guangfu Cancer Hospital between the period Jan 2021 to December 2023, total number of patients was 245. Patients were divided into two cohorts: DS of 107 and CRS of 138. Disease-free survival was the main predictor, while the others were considered secondary endpoints; these were overall survival, pathological complete response rate, postoperative complications, and R0 resection rate.

**Results:**

Disease-specific survival benefitted the CRS cohort relative to the DS cohort with a 2-year DFS of 76.81% compared to 65.42% (p= 0. 049). Median DFS also favored the CRS group (34. 7 months *vs* 28. 3 months, p = 0. 023). While not statistically significant, there was a trend towards improved OS in the CRS cohort (2-year OS rate: The results are as follows: 81. 16% versus 72. 90%, p=0.124. The CRS group had higher resection of R0 (92.8% *vs* 86.0%, p=0.082), and similarly, the local recurrence and distant metastases, although non-significant, were lower in this group.

**Conclusion:**

Locally advanced GI tract cancer appears to be improved from neoadjuvant-applied chemoradiotherapy followed by surgery rather than upfront surgery. Whereas, the trend about OS was in favor of the CRS approach, perhaps more time is required to observe these differences. The multimodal technique had reasonable presurgical toxicities and did not worsen the rate of postoperative complications. These findings support the consideration of neoadjuvant chemoradiotherapy can be recommended as a viable treatment approach for locally advanced GI tract cancers but future comparative prospective trials must be conducted to determine long-term survivals and quality of life patterns.

## 1 Introduction

Gastrointestinal (GI) tract cancers are a major health issue affecting millions of patients worldwide including a wide spectrum of neoplasms originating in organs of the digestive tract (1). These cancers are; esophageal cancer, gastric cancer, colorectal, and anal cancer; they contribute significantly to morbidity and mortality from cancers around the world (2). The therapy of locally advanced GI tract cancer has undergone dramatic changes in the last decades where emphasis has been given to the combination of surgery, chemotherapy, and radiotherapy (3). In the past, the operation has remained the mainstay of therapy for locally advanced GI tract cancers (4). But even assuming improvements in the surgical procedures and the management of the patients during the operation, the results of the patients who underwent surgery alone have been far from satisfactory, especially for those patients who have undergone surgery at an early stage of their diseases. Local recurrence and distant metastases continue to be major challenges in the management of colorectal cancer and the application of combined modality treatment that implies the integration of locoregional and systemic treatments for the comprehensive management of the disease (5).

Neoadjuvant therapy or pre-operative treatment aimed at performing chemotherapy and radiotherapy before resection of malignant tumors has been widely researched in GI tract cancers. This approach has several theoretical benefits on which it is based. First, neoadjuvant therapy can influence the tumor stage and size that can be removed by surgery fully (6). Second, it involves the possibility for the treatment of even tiny metastases, and thereby a chance to minimize the probability of the distant recurrence (7). Carboplatin and cisplatin platinum-containing regimens have become an important component of combined-modality treatment for GI tract cancers in the course of neoadjuvant therapy (8). These agents have shown effectiveness in many solid tumor types and there is evidence from many studies about synergy with other anticancer drugs as well as radiation (8). Carboplatin and cisplatin are the two main platinum-containing drugs used in chemotherapy (9); however, the choice between carboplatin and cisplatin can be influenced by tumor type, patient’s characteristics, side effects or toxicity profiles.

The incorporation of radiotherapy in neoadjuvant modalities has additionally illuminated other prospects of local tumor control (10). Improvements in radiation technology like IMRT and VMAT led to minimizing the dose to the healthy tissue and maximizing the dose to tumors (11). This has made it possible to establish new approaches in dose escalation and find other ways of increasing the efficiency of combined modality treatments. There are several reasons why chemotherapy and radiotherapy may be given concurrently in a neoadjuvant strategy. Radiosensitivity can also be seen in chemotherapy treatment where it functions as an enhancer of radiation therapy (12). Furthermore, the nonspecific cure of chemotherapy may also assist in eradicating micro metastases that may be out of the reach of localized radiotherapy (13). The use of these modalities in combination might enhance local and distant disease control. Despite the possible advantages of neoadjuvant chemoradiotherapy, its application in the treatment of locally advanced GI tract cancer has undergone a lot of research and controversy (14).

The effects that neoadjuvant therapy has on the surgery is another subject that has to be studied. While neoadjuvant therapy may enhance the chance of negative surgical margin (15), these techniques may also change the tissue planes and may thus be much more technique-demanding intra-operatively (16). To avoid postoperative complications and enhance the postoperative outcome it is significant to recognize and manage any possible negative consequences on it. However, the evaluation of the treatment response after providing neoadjuvant therapy has its strengths and weaknesses. Nevertheless, it remains difficult to predict and assess response to neoadjuvant therapy and ongoing efforts are being made to find better imaging modalities and molecular biomarkers (17). Other changes in GI tract cancer management are also applying the shift in oncology in general, including immunotherapy and target therapy (18). These techniques are not established in neoadjuvant protocols for GI tract cancers, although they have been utilized in several types of cancer (19). Current research is underway trying to add new agents to the established chemoradiotherapy regimens to enhance survival rates. The perceptual aspects or ‘‘quality of life’’ issues are today considered important determinants in the management of cancer. This is very crucial in tract malignancies, as the treatments we offer impact features of life such as peristalsis, ingestion, and defecation.

Therefore, the management of locally advanced GI tract cancers is dependent on several factors that call for a team effort from both medical professionals and patients (20). Multidisciplinary tumor boards containing surgeons, medical oncologists, radiation oncologists, radiologists, pathologists, and others are important in personalized medicine for cancer. Here some of the considerations are tumor location, stage, molecular characteristics, age, presence of the comorbidities, and, most importantly, preferences (21, 22).

While advanced research progresses in revealing fundamental ideas as to the progression, detailing cell reactions to therapies, and identification of individual patient attributes that define the likelihood of progression and responses, the ultimate aim is to enhance approaches to managing these intricate cancers. This study aims to provide useful information about these endeavors to try and achieve better survival and quality of life for those with locally advanced cancer of the GI tract.

## 2 Materials and methods

### 2.1 Study design and population

This study was a retrospective analysis conducted at Zhejiang Jinhua Guangfu Cancer Hospital. We reviewed the medical records of patients diagnosed with locally advanced gastrointestinal tract cancer who underwent treatment between January 2021, and December 2023. The study protocol was approved by the Institutional Review Board of Zhejiang Jinhua Guangfu Cancer Hospital, and the requirement for informed consent was waived due to the retrospective nature of the study.

A total of 245 patients met the inclusion criteria and were included in the analysis. The inclusion criteria were as follows: Age ≥ 18 years, histologically confirmed locally advanced gastrointestinal tract cancer (including esophageal, gastric, colorectal, and anal cancers), Clinical stage II-III according to the 8^th^ edition of the American Joint Committee on Cancer (AJCC) TNM staging system, Adequate organ function. Exclusion criteria included: Distant metastases at the time of diagnosis, Previous history of malignancy within the past 5 years, Contraindications to surgery or chemotherapy, and Incomplete medical records.

### 2.2 Treatment groups

Patients were divided into two cohorts based on their treatment approach:

1. Direct Surgery (DS) cohort (n = 107): Patients who underwent upfront surgery without neoadjuvant therapy.
2. Chemoradiotherapy followed by Surgery (CRS) cohort (n = 138): Patients who received neoadjuvant platinum-based chemotherapy followed by radiotherapy and then surgery.

### 2.3 Treatment protocols

#### 2.3.1 Direct surgery cohort

Patients in this group underwent standard surgical procedures appropriate for their specific gastrointestinal cancer type and location. The surgical approaches included: Esophagectomy for esophageal cancer, Gastrectomy (total or subtotal) for gastric cancer, Colectomy or low anterior resection for colorectal cancer, and Abdominoperineal resection for anal cancer. For anal cancer patients in our cohort, surgery was performed following neoadjuvant chemoradiotherapy, in accordance with our institutional protocol. We acknowledge that this approach differs from the widely accepted standard of care, which generally consists of definitive chemoradiotherapy without surgery. The extent of lymph node dissection was determined based on the tumor location and stage. All surgeries were performed by experienced surgical oncologists following standardized protocols.

#### 2.3.2 Chemoradiotherapy followed by surgery cohort

Patients in this group received a multimodal treatment approach consisting of:

#### 2.3.3 Neoadjuvant chemotherapy

Patients received either carboplatin or cisplatin-based chemotherapy regimens. The choice between carboplatin and cisplatin was made by the treating oncologist based on individual patient factors such as renal function, comorbidities, and potential toxicity profiles. Each patient received only one platinum drug throughout their treatment course. Common regimens included, For upper gastrointestinal cancers: a) Carboplatin (AUC 5) on day 1 + 5-fluorouracil (5-FU) 1000 mg/m2/day continuous infusion on days 1-4, every 3 weeks for 2–3 cycles OR b) Cisplatin 75 mg/m2 on day 1 + 5-FU 1000 mg/m2/day continuous infusion on days 1-4, every 3 weeks for 2–3 cycles. For colorectal and anal cancers: a) Carboplatin (AUC 5) on day 1 + capecitabine 1000 mg/m2 twice daily on days 1-14, every 3 weeks for 2–3 cycles OR b) Cisplatin 75 mg/m2 on day 1 + capecitabine 1000 mg/m2 twice daily on days 1-14, every 3 weeks for 2–3 cycles.

#### 2.3.4 Radiotherapy

Following neoadjuvant chemotherapy, patients underwent radiotherapy. The radiation dose and fractionation were tailored to the specific cancer type and location. For example, for Esophageal cancer it was 41.4-50.4 Gy in 23–28 fractions, for Gastric cancer it was 45 Gy in 25 fractions, for Rectal cancer it was 45-50.4 Gy in 25–28 fractions, and for Anal cancer, it was 50.4–54 Gy in 28–30 fractions.

Intensity-modulated radiation therapy (IMRT) or volumetric modulated arc therapy (VMAT) techniques were used to deliver the prescribed dose while minimizing exposure to surrounding healthy tissues.

#### 2.3.5 Surgery

Approximately 6–8 weeks after completing chemoradiotherapy, patients underwent surgical resection following the same principles as the DS cohort. The timing of surgery was determined based on the patient’s recovery from neoadjuvant therapy and restaging results. Specifically, all patients with anal cancer in the CRS cohort underwent abdominoperineal resection following completion of chemoradiotherapy, as per our institutional multidisciplinary treatment strategy.

### 2.4 Outcome measures

The primary outcome measure was Disease-Free Survival (DFS), defined as the time from the date of surgery (for both cohorts) to the date of first recurrence, death from any cause, or last follow-up, whichever occurred first.

Secondary outcome measures included:

1. Overall Survival (OS): Time from the date of diagnosis to the date of death from any cause or last follow-up.
2. Pathological complete response rate in the CRS cohort.
3. Postoperative complication rates.
4. R0 resection rates (negative surgical margins). R0 resection was defined as microscopically negative resection margins (proximal, distal, and circumferential), with no viable tumor cells at the inked margin.

### 2.5 Statistical analysis

All statistical analyses were performed using SPSS version 26.0 (IBM Corp., Armonk, NY, USA). Continuous variables were expressed as mean ± standard deviation or median (interquartile range) depending on the distribution of data. Categorical variables were presented as frequencies and percentages. A two-sided p-value < 0.05 was considered statistically significant for all analyses. Additionally, multivariable Cox proportional hazards regression was performed to adjust for potential confounders, including clinical stage, age, and performance status, with disease-free survival and overall survival as dependent variables. A p-value < 0.05 was considered statistically significant.

Given the exploratory design, multiple subgroup analyses were performed. No formal correction for multiple testing was applied in the primary analyses; however, Bonferroni-adjusted p-values are reported in **Supplementary Table 2** for transparency. DFS was defined from the date of surgery to recurrence or death. As a sensitivity analysis, DFS was also calculated from the date of diagnosis to assess potential lead-time bias.

### 2.6 Sample size

The sample size was determined based on the available patient population treated at Zhejiang Jinhua Guangfu Cancer Hospital during the specified period (2021-2023). With 245 patients (107 in the DS cohort and 138 in the CRS cohort).

### 2.7 Ethical considerations

The study protocol was approved by the Institutional Review Board of Zhejiang Jinhua Guangfu Cancer Hospital (Approval No. ZJHGFCH-2024-001). The study was conducted in accordance with the Declaration of Helsinki. Given its retrospective design and use of anonymized patient data, the requirement for individual informed consent was waived by the ethics committee.

## 3 Results

### 3.1 Patient characteristics

This retrospective analysis included 245 patients with locally advanced gastrointestinal tract cancer. The Direct Surgery (DS) cohort comprised 107 patients, while the Chemoradiotherapy followed by Surgery (CRS) cohort included 138 patients. There were no significant differences between the two groups in terms of age, sex, BMI, ECOG performance status, or cancer type distribution (**Table 1**).

**Table 1:** Patient and treatment characteristics and comparison of demographic and tumor-related parameters of the patients.

Characteristic	Direct surgery (DS) cohort (n=107)	Chemoradiotherapy followed by surgery (CRS) cohort (n=138)	p-value
Age, years (mean ± SD)	62.5 ± 11.3	60.8 ± 10.7	0.230
Sex, n (%)			0.506
- Male	65 (60.7%)	78 (56.5%)	
- Female	42 (39.3%)	60 (43.5%)	
BMI, kg/m² (mean ± SD)	24.3 ± 3.8	23.9 ± 4.1	0.435
ECOG Performance Status, n (%)			0.777
0	45 (42.1%)	62 (44.9%)	
-1	52 (48.6%)	61 (44.2%)	
-2	10 (9.3%)	15 (10.9%)	
Cancer Type, n (%)			0.866
- Esophageal	28 (26.2%)	40 (29.0%)	
- Gastric	35 (32.7%)	48 (34.8%)	
- Colorectal	38 (35.5%)	42 (30.4%)	
- Anal	6 (5.6%)	8 (5.8%)	
Clinical Stage, n (%)			0.035*
- II	49 (45.8%)	45 (32.6%)	
- III	58 (54.2%)	93 (67.4%)	
Type of Surgery, n (%)			0.866
-Esophagectomy	28 (26.2%)	40 (29.0%)	
-Gastrectomy	35 (32.7%)	48 (34.8%)	
-Colectomy/Low anterior resection	38 (35.5%)	42 (30.4%)	
-Abdominoperineal resection	6 (5.6%)	8 (5.8%)	
Operative time, minutes (mean ± SD)	245 ± 62	278 ± 71	0.001*
Blood loss, mL (median [IQR])	280 [150–450]	320 [180–520]	0.038*
R0 resection rate , n (%)	92 (86.0%)	128 (92.8%)	0.082
Postoperative complications, n (%)	28 (26.2%)	42 (30.4%)	0.463

*Statistically significant (p < 0.05) SD, Standard Deviation; IQR, Interquartile Range.

However, the CRS cohort had a significantly higher proportion of patients with clinical stage III disease compared to the DS cohort (67.4% *vs*. 54.2%, p=0.035). This difference in staging distribution should be considered when interpreting the outcomes.

### 3.2 Treatment outcomes

Surgical Outcomes: The CRS cohort had longer operative times (278 ± 71 min *vs*. 245 ± 62 min, p=0.001) and higher intraoperative blood loss (median 320 mL *vs*. 280 mL, p=0.038) compared to the DS cohort. Despite these differences, there was no significant difference in postoperative complication rates between the two groups (30.4% for CRS *vs*. 26.2% for DS, p=0.463). The R0 resection rate was higher in the CRS group, although this difference did not reach statistical significance (92.8% *vs*. 86.0%, p=0.082). A pathological complete response (pCR) was achieved in 20 of 138 patients (14.5%) who received neoadjuvant chemoradiotherapy followed by surgery.

### 3.3 Survival outcomes

With a median follow-up of 28 months (range 12-48) in the DS cohort and 30 months (range 12-48) in the CRS cohort, the CRS group demonstrated improved survival outcomes (**Table 2**):

1. Disease-Free Survival (DFS): The CRS cohort had a significantly higher 2-year DFS rate compared to the DS cohort (76.81% *vs*. 65.42%, p=0.049). The median DFS was also significantly longer in the CRS group (34.7 months *vs*. 28.3 months, p=0.023).
2. Overall Survival (OS): Although not statistically significant, there was a trend towards improved OS in the CRS cohort. The 2-year OS rate was 81.16% in the CRS group compared to 72.90 in the DS group (p=0.124). The median OS was 41.2 months in the CRS group versus 36.5 months in the DS group (p=0.062).
3. In multivariable Cox regression analysis adjusted for clinical stage, the CRS cohort remained significantly associated with improved disease-free survival (HR: 0.73, 95% CI: 0.54–0.98, p = 0.038). The trend toward improved overall survival in the CRS group persisted after adjustment, although it did not reach statistical significance (HR: 0.82, 95% CI: 0.61–1.11, p = 0.190).
4. Exploratory subgroup analyses by cancer type are presented in **Supplementary Table 1**. Although the study was not powered to detect statistically significant differences within individual cancer types, the CRS group consistently showed a trend toward improved disease-free survival across esophageal, gastric, colorectal, and anal cancers, with the strongest effect observed in rectal and esophageal cancer patients.
5. When DFS was defined from the date of surgery, CRS was associated with significantly improved DFS compared with DS (HR: 0.71, 95% CI: 0.52–0.97, unadjusted p = 0.049; Bonferroni-adjusted p = 0.245). In a sensitivity analysis defining DFS from the date of diagnosis, the CRS group continued to demonstrate a trend toward improved DFS, although statistical significance was attenuated (HR: 0.77, 95% CI: 0.57–1.03, unadjusted p = 0.071; Bonferroni-adjusted p = 0.355) (**Supplementary Table 2**).
6. We further compared outcomes between patients treated with carboplatin- versus cisplatin-based neoadjuvant regimens. Two-year DFS was 75.0% in the carboplatin group and 78.0% in the cisplatin group (p = 0.62), while 2-year OS was 80.0% versus 82.5% (p = 0.78) **Supplementary Table 3**.

**Table 2:** Local recurrence, distant metastases, overall survival, and disease-free survival rates of the patients.

Outcome	Direct Surgery (DS) Cohort (n=107)	Chemoradiotherapy followed by Surgery (CRS) Cohort (n=138)	p-value
Median follow-up, months (range)	28 (12-48)	30 (12-48)	0.624
Local recurrence, n (%)	18 (16.8%)	14 (10.1%)	0.124
Distant metastases, n (%)	25 (23.4%)	22 (15.9%)	0.143
2-year Overall Survival rate (%)	72.90%	81.16%	0.124
2-year Disease-Free Survival rate (%)	65.42%	76.81%	0.049*
Median Overall Survival, months (95% CI)	36.5 (32.8-40.2)	41.2 (37.9-44.5)	0.062
Median Disease-Free Survival, months (95% CI)	28.3 (24.6-32.0)	34.7 (31.2-38.2)	0.023*

*Statistically significant (p < 0.05) CI, Confidence Interval.

### 3.4 Recurrence patterns

The CRS cohort showed lower rates of both local recurrence (10.1% *vs*. 16.8%, p=0.124) and distant metastases (15.9% *vs*. 23.4%, p=0.143) compared to the DS cohort, although these differences did not reach statistical significance.

### 3.5 Toxicity

**Tables 3**, **4** detail the acute toxicities observed during chemoradiotherapy and adjuvant chemotherapy in the CRS cohort. The most common grade 3–4 toxicities during chemoradiotherapy were neutropenia (5.8%), fatigue (5.8%), and nausea (5.1%). During adjuvant chemotherapy, the most frequent grade 3–4 toxicities were neutropenia (5.1%), fatigue (4.3%), and anemia (2.9%). No grade 4 toxicities were observed for most adverse events, indicating that the treatment was generally well-tolerated.

**Table 3:** The acute toxicity encountered during the chemoradiotherapy period for all patients.

Toxicity	Grade 1 n (%)	Grade 2 n (%)	Grade 3 n (%)	Grade 4 n (%)
Hematological				
- Anemia	28 (20.3%)	15 (10.9%)	5 (3.6%)	0 (0%)
- Neutropenia	35 (25.4%)	22 (15.9%)	8 (5.8%)	2 (1.4%)
- Thrombocytopenia	20 (14.5%)	10 (7.2%)	3 (2.2%)	0 (0%)
Gastrointestinal				
- Nausea	45 (32.6%)	25 (18.1%)	7 (5.1%)	0 (0%)
- Vomiting	30 (21.7%)	18 (13.0%)	5 (3.6%)	0 (0%)
- Diarrhea	25 (18.1%)	15 (10.9%)	4 (2.9%)	0 (0%)
- Mucositis	22 (15.9%)	12 (8.7%)	3 (2.2%)	0 (0%)
Other				
- Fatigue	50 (36.2%)	30 (21.7%)	8 (5.8%)	0 (0%)
- Dermatitis	35 (25.4%)	20 (14.5%)	5 (3.6%)	0 (0%)
- Nephrotoxicity	15 (10.9%)	8 (5.8%)	2 (1.4%)	0 (0%)

**Table 4:** The acute toxicity related to the adjuvant chemotherapy period for the patients in the chemoradiotherapy + chemotherapy (CRT+CT) group.

Toxicity	Grade 1 n (%)	Grade 2 n (%)	Grade 3 n (%)	Grade 4 n (%)
Hematological				
- Anemia	20 (14.5%)	12 (8.7%)	4 (2.9%)	0 (0%)
- Neutropenia	25 (18.1%)	18 (13.0%)	7 (5.1%)	1 (0.7%)
- Thrombocytopenia	15 (10.9%)	8 (5.8%)	2 (1.4%)	0 (0%)
Gastrointestinal				
- Nausea	30 (21.7%)	20 (14.5%)	5 (3.6%)	0 (0%)
- Vomiting	22 (15.9%)	15 (10.9%)	3 (2.2%)	0 (0%)
- Diarrhea	18 (13.0%)	10 (7.2%)	2 (1.4%)	0 (0%)
- Mucositis	15 (10.9%)	8 (5.8%)	1 (0.7%)	0 (0%)
Other				
- Fatigue	35 (25.4%)	22 (15.9%)	6 (4.3%)	0 (0%)
- Neuropathy	20 (14.5%)	12 (8.7%)	3 (2.2%)	0 (0%)
- Nephrotoxicity	10 (7.2%)	5 (3.6%)	1 (0.7%)	0 (0%)

This retrospective analysis suggests that a multimodal approach of neoadjuvant chemoradiotherapy followed by surgery may offer improved disease-free survival compared to upfront surgery in patients with locally advanced gastrointestinal tract cancer. While there was a trend towards improved overall survival with the multimodal approach, this difference did not reach statistical significance. The neoadjuvant approach was associated with acceptable toxicity profiles and did not significantly increase postoperative complications.

## 4 Discussion

This retrospective study showed the respective survivals between the use of DS and neo-chemoradiotherapy with subsequent CRS in treating localized gastrointestinal tract malignancies. The examination of 245 patients (107 in the DS cohort and 138 in the CRS cohort) who received Zhejiang Jinhua Guangfu Cancer Hospital treatment from 2021 and 2023. The target patient population consisted of patients diagnosed with different types of GI Cancer, such as esophageal, gastric, colon, and anal cancer. There was no significant difference in the cancer distribution for the two groups such that each comparison was balanced. However, there is a noteworthy difference between the CRS and DS cohort regarding clinical stage: a higher proportion of patients in the CRS cohort were diagnosed with clinical stage III (67. 4% *vs*. 54. 2%, p = 0. 035). The importance of the understanding of the results is the difference in staging distribution, which indicates the fact that patients with more developed diseases were treated with neoadjuvant therapy. This approach is consistent with treatment plans in which there is a combination of therapy and medication for higher stages of GI cancers. The CRS group needed longer operative time than the DS group (278 ± 71 min *vs* 245 ± 62 min, p = 0.001) together with higher intraoperative blood loss; (Median, 320 mL *vs* 280 mL, p = 0.038). These results are comparable with the studies that have established that there is enhanced surgical invasiveness after neoadjuvant treatment. For example (23, 24), Discussed neoadjuvant chemoradiotherapy in rectal cancer where patients had similar prolongation of their operative time and a similar increase in blood loss as patients not treated with preoperative chemoradiotherapy.

However, it has to be noted that there was no substantial difference in intraoperative parameters between the two groups, except for the duration of operation, which was shorter in the case of DS (278 ± 71 min *vs*. 245 ± 62 min, p<0. 001). This leaves an impression that neoadjuvant therapy did not up the ante in terms of surgical morbidity thus it is significant in the safety compliance of the patients as well as their quality of life. This finding agrees with a large retrospective study by (25) on esophageal cancer where they also did not report a significant increase in postoperative complications following neoadjuvant chemoradiotherapy. Although this did not reach statistical significance, the clinically relevant increase of the R0 resection rate in the CRS group (92. 8% *vs*. 86. 0%, p=0. 082). Furthermore, the percentage of T3/4 tumors did not differ significantly between both groups. Surgical margin status is one of the important predictors of long-term oncological results. The increased R0 resection rate observed in the CRS cohort fully corresponds with the results of other investigations of various GICTs. For instance, a study of esophageal cancer done by (26) that involved a randomized controlled trial showed that a combination of neoadjuvant chemoradiotherapy and surgery enhanced R0 resection as compared to surgery alone registering 92% as opposed to 69% p<0.001 Another study that gave short-term adjuvant chemotherapy after CRS was able to demonstrate improved DFS in their CRS population. DFS, with a 2-year DFS rate of 76. 81% in the CRS group *vs* 65. 42% in the surgery group, p=0. 049, median DFS of 34. 7 months in the CRS group *vs* 28. 3 months in the surgery group, p=0.023. These results strengthen the argument that neoadjuvant chemoradiotherapy should be more frequently applied in locally advanced gastrointestinal cancers. The increase in DFS noted in this study has been registered in several major randomized controlled trials as well as meta-analyses in various kinds of gastrointestinal cancers. For example, in a CROSS trial randomized patients with esophageal cancer (27) observed a statistically significant increase in DFS with neoadjuvant chemoradiotherapy. Likewise, in rectal cancer, the German CAO/ARO/AIO-94 trial (27) confirmed significantly better DFS in patients with preoperative chemoradiation than in those with postoperative treatment.

In the present study, a trend toward a better overall survival was observed in the CRS group with a 2-year overall survival of 81.16% compared to 72. 90% in the DS group, the difference was nevertheless not statistically significant (p=0. 124). Similar trends were noted where the median overall survival (OS) was also numerically better in the CRS group (41. Even though we found a statistically significant improvement in DFS, we did not find a statistically significant improvement in OS, a situation that is not strange when analyzing the results of neoadjuvant treatment in gastrointestinal cancers. This pattern has been established in multiple trials among others being the CROSS trial in esophageal cancer whereby the up. The study also reported reduced rates of local recurrence in the CRS group (10. 1% *vs* 16. 8%; P = 0.124) and distant metastases (15. 9% *vs* 23. 4%; P = 0.143) as compared to the DS group but these were not statistically significant. Such findings indicate that neoadjuvant chemoradiotherapy may assist in the management of locoregional and systemic diseases hence improved DFS noted in the CRS group.

The trend of less local recurrence in the CRS group is complemented by several works dedicated to various types of gastrointestinal cancers. For instance in rectal cancer, a Dutch TME trial (28, 29) established a considerable decrease in local relapse in patients who received preoperative radiotherapy. Likewise in Gastric cancer (30), reported better local control with perioperative chemotherapy. There are some potential decreases in distant metastases in this work; it seems that neoadjuvant therapy has not only the local effect on tumor bulk but also the systemic effect. Neoadjuvant therapy is thought to be advantageous for reasons, including control of micrometastases and enhanced drug delivery to the primary tumor site owing to vascular permeability, which gains support from this study.

The study also offered the investigators explicit findings of acute toxicities during chemoradiotherapy and adjuvant chemotherapy in the CRS group. The incidence of grade 3–4 toxicities during chemoradiotherapy was neutropenia at 5.8%, and fatigue at 5. 8%, and nausea 5. 1%. When receiving adjuvant chemotherapy, the most common serious toxicity that occurred in the first cycle was neutropenia at 5.1%, fatigue 4. At 3%, and anemia 2.9%. The above-mentioned toxicity profiles are quite in tandem with other published reports of neoadjuvant chemoradiotherapy in gastrointestinal cancers. For example, the CROSS trial of chemoradiotherapy for esophageal cancer revealed similar toxicities of hematologic and gastrointestinal origins. The lack of grade 4 toxicities for most AEs in this study raises the idea that the neoadjuvant approach to the presented treatment regimen was spared from critical side effects. An important limitation of our study is that patients with anal cancer underwent surgery after neoadjuvant chemoradiotherapy, whereas the internationally accepted standard of care is definitive chemoradiotherapy alone. This reflects institutional practice at our center, and therefore our findings for anal cancer patients should be interpreted with caution in the context of existing global guidelines. A major limitation of this study is the imbalance in baseline clinical stage between cohorts, with more stage III patients in the CRS group. Although we performed multivariable regression to adjust for stage, residual confounding cannot be excluded. Future multicenter studies using propensity score matching or randomized trial designs are warranted to minimize selection bias. The study has however a few important limitations that merit consideration, among them is the fact that the long-term toxicities as well as the quality-of-life issues were not assessed within the study. It should also be noted that in our study, rectal cancer patients received carboplatin or cisplatin rather than oxaliplatin, which is currently the standard platinum agent recommended in international guidelines (31, 32). This reflects institutional practice but may limit the comparability of our findings to centers using oxaliplatin-based total neoadjuvant therapy protocols. The median follow-up of 28–30 months is relatively short, limiting the ability to draw firm conclusions about long-term overall survival, especially in gastric and colorectal cancers. This study possesses several advantages: a rather large patient cohort, multiple types of gastrointestinal cancer, a clear description of surgical results, and toxic effects. Performing the surgeries with standardized treatment plans and qualified surgical teams contributes to the credibility of the results. However, there are also important limitations that should be mentioned: selective inclusion of patients that created the risk of selection bias and confounding factors. The findings may not extend to other hospitals or clients. Although including multiple gastrointestinal cancers gives a wide view of the results, it may obscure the effects of cancer-specific treatment. The median follow-up of 28–30 months might have been insufficient to assess the survival rate, especially in patients with better prognosis about their cancer type. In this work, a posteriori, there is argumentation towards the use of neoadjuvant chemoradiotherapy and then surgery for locally advanced gastrointestinal tract cancers. The significant improvement in disease-free survival, the trend towards better overall survival, and the acceptable toxicity profile suggest that this multimodal approach may offer benefits over upfront surgery alone.

## 5 Conclusion

The results of this study show the use of neoadjuvant chemoradiotherapy and then surgery as a viable approach to the management of locally advanced GI tract cancer. This approach seems to provide better disease control and yet results in little or no increase in the morbidity from the treatment. However, long-term prospective, randomized studies with better selection criteria, extended follow-up, and better definition of disease and quality of life parameters outcome need to be performed. Also, further study should involve the evaluation of patient populations that are most likely to benefit from this multimodal system and analysis of improvements to the neoadjuvant regime that can be made, including target therapies or immunotherapy.

## Data Availability

The original contributions presented in the study are included in the article/**Supplementary Material**. Further inquiries can be directed to the corresponding authors.
