# Supplementary material for: Surgery versus neoadjuvant chemoradiotherapy followed by surgery in locally advanced gastrointestinal tract cancers
Source: Front Oncol. 2025 Sep 10;15:1631549. doi: 10.3389/fonc.2025.1631549 (PMC12457145; doi:10.3389/fonc.2025.1631549)
Supplement: Supplementary file 1 [file Table1.docx]

**Supplementary Tables**.

**Supplementary Table 1: Exploratory subgroup analyses of disease-free survival (DFS) by cancer type**

| **Cancer Type** | **Group** | **n** | **2-year DFS (%)** | **HR (95% CI) CRS vs. DS** | **p-value** |
| --- | --- | --- | --- | --- | --- |
| Esophageal | DS | 28 | 64.3 |  |  |
|  | CRS | 40 | 75 | 0.74 (0.46–1.21) | 0.23 |
| Gastric | DS | 35 | 63 |  |  |
|  | CRS | 48 | 77.1 | 0.69 (0.43–1.10) | 0.11 |
| Colorectal | DS | 38 | 68.4 |  |  |
|  | CRS | 42 | 79.2 | 0.70 (0.42–1.18) | 0.18 |
| Anal | DS | 6 | 50 |  |  |
|  | CRS | 8 | 75 | 0.55 (0.18–1.66) | 0.29 |

**Supplementary Table 2. Sensitivity analysis of DFS definitions and correction for multiple comparisons**

| **DFS Start Point** | **Group** | **2-year DFS (%)** | **HR (95% CI) CRS vs. DS** | **Unadjusted p-value** | **Bonferroni-adjusted p-value*** | **Interpretation** |
| --- | --- | --- | --- | --- | --- | --- |
| **From Surgery Date** | DS | 65.4 |  |  |  |  |
|  | CRS | 76.8 | 0.71 (0.52–0.97) | 0.049 | 0.245 | Significant before correction; trend after adjustment. |
| **From Diagnosis Date** | DS | 64 |  |  |  |  |
|  | CRS | 74.2 | 0.77 (0.57–1.03) | 0.071 | 0.355 | Non-significant; benefit trend remains. |

## ****Supplementary Table 3. Comparison of outcomes between carboplatin- and cisplatin-based regimens in rectal and anal cancer patients****

| **Outcome** | **Carboplatin-based (n = 72)** | **Cisplatin-based (n = 66)** | **p-value** | **Interpretation** |
| --- | --- | --- | --- | --- |
| 2-year DFS (%) | 75 | 78 | 0.62 | No significant DFS difference between regimens. |
| 2-year OS (%) | 80 | 82.5 | 0.78 | No significant OS difference. |
